# A Novel Humanized Anti-Abrin A Chain Antibody Inhibits Abrin Toxicity *In Vitro* and *In Vivo*

**DOI:** 10.3389/fimmu.2022.831536

**Published:** 2022-02-04

**Authors:** Jingyi Peng, Jiaguo Wu, Ning Shi, Hua Xu, Longlong Luo, Jing Wang, Xinying Li, He Xiao, Jiannan Feng, Xia Li, Lihui Chai, Chunxia Qiao

**Affiliations:** ^1^Joint National Laboratory for Antibody Drug Engineering, The First Affiliated Hospital, School of Medicine, Henan University, Kaifeng, China; ^2^State key Laboratory of Toxicology and Medical Countermeasures, Institute of Pharmacology and Toxicology, Academy of Military Medical Sciences, Academy of Military Sciences, Beijing, China; ^3^Department of Anatomy, School of Basic Medical Sciences of Dali University, Dali, China; ^4^Cancer Research Institute, School of Basic Medical Science, Central South University, Changsha, China; ^5^School of Pharmacy, Henan University, Kaifeng, China

**Keywords:** abrin, humanization, neutralizing antibody, protective effect, computer-aided design

## Abstract

Abrin, a type-II ribosome inactivating protein from the seed of *Abrus precatorius*, is classified as a Category B bioterrorism warfare agent. Due to its high toxicity, ingestion by animals or humans will lead to death from multiple organ failure. Currently, no effective agents have been reported to treat abrin poisoning. In this study, a novel anti-abrin neutralizing antibody (S008) was humanized using computer-aided design, which possessed lower immunogenicity. Similar to the parent antibody, a mouse anti-abrin monoclonal antibody, S008 possessed high affinity and showed a protective effect against abrin both *in vitro* and *in vivo*, and protected mice that S008 was administered 6 hours after abrin. S008 was found that it did not inhibit entry of abrin into cells, suggesting an intracellular blockade capacity against the toxin. In conclusion, this work demonstrates that S008 is a high affinity anti-abrin antibody with both a neutralizing and protective effect and may be an excellent candidate for clinical treatment of abrin poisoning.

## Introduction

Abrin toxin (AT) is a type II ribosome-inactivating protein (RIP) extracted from the seeds of the *Abrus precatorius* plant (rosary pea, jequirity bean), inhibits protein synthesis and induce apoptosis. Compared with ricin, another RIP, abrin is about 75 more toxic, and is classified as a Category B biological warfare agents in the U.S. due to its widespread source, ease of preparation, and lethality (1, 2). Abrin is a heterodimeric protein with the molecular weight of about 63 kDa, consisting of an A chain with N-glycosidase activity and a galactose-specific lectin B chain (3, 4). Abrin B chain (~33 kDa) is a lectin that binds to a β-D-galactoside moiety on the cell surface and mediating the internalization/endocytosis of the whole toxin into the host cell. There are four isoforms abrin-a, abrin-b, abrin-c and abrin-d, of which abrin-a and abrin-d are the most toxic. Abrin-b and c show weak lectin activity of B chains (5). After entering the cell, the A chain (~30 kDa) can hydrolyze the N-glycosidic bond of the 28S ribosomal RNA of eukaryotic cells and catalyze depurination, leading to inactivation of ribosomes and inhibition of protein synthesis, thereby causing cell apoptosis (6, 7). Clinically, abrin is considered to be an immunotoxin that can target cancer cells and can be delivered to tumor sites by coupling it with anti-tumor drugs (8).

Possible exposure to abrin is through injection, inhalation and mastication of seeds. After exposure, it can take several hours to days to develop symptoms, including nausea, vomiting, anorexia, cyanosis, functional failure of liver and kidney; cause of death is usually multi-organ failure. At present, the main clinical treatment is supportive care (9). Prevention of abrin poisoning by passive immunization has been reported on numerous occasions. In mice, generation of protective antibodies can be induced with a sub-lethal dose of abrin (10, 11). Recombinant abrin A chain (12–15) or the weaker abrin B chain (16–19), can also induce anti-abrin antibody production. Up to now, only two neutralizing antibodies against Abrin poisoning have been reported, D6F10 and A7C4, but neither has entered clinical trials or been used directly in humans. D6F10 is an IgG-class antibody that was produced against recombinant abrin A chain through hybridoma technology (12). A similar method was used to screen A7C4, a monoclonal antibody against Abrin A chain (20). D6F10 and A7C4 showed satisfactory neutralization activity against abrin *in vitro* as well as *in vivo*.

Because of their safety and effectiveness, monoclonal antibodies are widely used in clinical treatments for various kinds of diseases. However, mouse-derived antibodies that enter the human body will be recognized as exogenous proteins, and the immune system will respond to induce a human anti-mouse antibody (HAMA) reaction. Since heterologous proteins are removed quickly, they have a short half-life, which reduced their effectiveness. Due to the limitations of mouse-derived antibodies in clinical application, people often humanize them using molecular biology methods. Recently, with the development of bioinformatics and recombinant antibody technologies, dozens of humanized antibodies have been approved for clinical treatment. Mechaly et al. (21) constructed an immune scFv phage-display library by immunizing rabbits with abrin and isolated a panel of monoclonal antibodies, then modified them to obtain a chimeric rabbit-human scFv-Fc antibody, which could treat lung injury caused by abrin poisoning in mice. In contrast, our antibody replaces the mouse-derived codon bias, which results in higher humanization scores as it increases the acceptability of receptor immune system while ensuring the stability, specificity and affinity of the antibody (22). Here, based on the 3-dimentional structure of mouse anti-abrin hybridoma 10D8, using homology modelling, we prepared a novel anti-abrin neutralizing humanized antibody (S008). We also evaluated the neutralizing activity of S008 *in vitro* and *in vivo* in order to test its potential as an effective therapy that can treat abrin poisoning in humans.

## Materials and Methods

### Reagents

Abrin toxin was prepared by Biointron Biological Inc. (Beijing). CCK-8 detection kits were purchased from Dojindo (Japan). TNT^®^ Coupled Reticulocyte Lysate Systems were purchased from Promega (Cat. No.: L4610). Dulbecco’s modified Eagle medium (DMEM) (Cat. No.: 11965-092), RPMI 1640 media (Cat. No.: 61870-036) and fetal bovine serum (FBS) (Cat. No.: 10438-034) were purchased from ThermoFisher (U.S.). pFRT-KIgG1, a full-IgG expression plasmid fused with the constant regions of IgG1 heavy chain and kappa chain, was prepared in our lab based on the pcDNA5-FRT plasmid (ThermoFisher, Cat. No.: V601020). ExpiCHO-S cells and ExpiCHO™ Expression System (Cat. No.: A29133) were purchased from Gibco (ThermoFisher, U.S.). HiTrap MabSelect SuRe was purchased from Cytiva (U.S.). Alexa-FluorTM 488 and 594 were prepared by Jiaxuan Biotech (Beijing, China). TruStain FcXTM (anti-mouse CD16/32 and anti-Human IgG Fc receptors) were procured from Biolegend. All plastic-ware was procured from NuncTM (Denmark). All other chemicals were from commercial sources and of analytical grade.

### Cell Culture

Jurkat (ATCC TIB-152) were cultured in RPMI 1640 medium supplemented with 100 units/mL penicillin, 100 units/mL streptomycin and 10% heat-inactivated FBS. Vero (ATCC CCL-81) cells were cultured in DMEM medium supplemented with 100 units/mL penicillin, 100 units/mL streptomycin and 10% heat-inactivated FBS. Cells were seeded onto 10-cm cell culture dishes (NuncTM, Den-mark), placed in a humidified incubator (Thermo, Waltham, MA, USA) at 37°C in 5% CO_2_ and passaged every 2-3 days. Adherent cultures were digested with 0.25% trypsin–EDTA. ExpiCHO-S cells were cultured in serum-free and protein-free ExpiCHO™ Expression Medium and incubated in Polycarbonate Erlenmeyer shake flasks in a 37°C incubator with 80% humidity and 8% CO_2_ on an orbital shaker platform. Cells were typically passaged every 3-4 days.

### Humanization

To decrease the immunogenicity of the murine antibody 10D8 derived from hybridoma technology, humanization was carried out using http://www.abysis.org. 3-D structures of the murine and humanized antibody, including the variable domains of the heavy chain and light chain, were constructed. Fv fragments were modeled using computer guided homology modeling. 3-D structures between 10D8 and humanized antibody S008 were compared using the structural superimposition method.

### Antibody Expression and Purification

Variable sequences of S008 heavy chain and light chain were subcloned into the full-length IgG1 expression vector pFRT-KIgG1, to generate plasmid S008-pFRT-KIgG1, which was transfected into ExpiCHO-S cells. At 18 h after transfection, ExpiFectamine™ CHO Enhancer was added. Humanized antibody S008 was secreted into the culture medium. After 8-9 days, supernatant was collected, and S008 was purified using HiTrap MabSelect SuRe. The purity of purified antibodies was analyzed using size exclusion chromatography HPLC Columns (SEC-HPLC). The chromatographic experiment was done with a flow rate of 1 mL/min, and the injection volume was 20 μL while the column temperature was 35°C. The absorption at 280 nm was detected for an acquisition time of 15 min to obtain the chromatographic analysis data. For fluorescence-related analysis, the purified antibodies were labeled with Alexa-594 and Alaxa-488, respectively, which was supplied by Jiaxing Biologicals using Alexa Fluor Protein Labeling Kits, followed by antibody purification by Sephadex G-25.

### Enzyme-Linked Immunosorbent Assay

A 2-μg/mL of abrin (100 μL/well) was coated overnight at 4°C in enzyme-linked immunosorbent assay (ELISA) plate wells (Nunc™, Denmark). All tests were performed in duplicate. Wells were washed three times with PBS containing 0.2% Tween-20 and blocked with 200 μL of 4% PBS-milk for 2 h at 37°C. After washing, 100 μL of gradient-diluted S008 (10 μg/mL to 100 pg/mL) was added to the well and incubated for 1 h at 37°C. After washing, 100 μL of horse radish peroxidase (HRP)-conjugated goat anti-human IgG (1:4000) was added to the wells, incubated for 45 min at RT, and the enzymatic reaction was developed with TMB substrate. Termination of the reaction was performed by adding 100 μL/well of 1mol/L H_2_SO_4_. Plates were measured at 450 nm in a microtiter plate reader. The concentration for 50%of maximal effect was defined as EC50. EC50 values were calculated using GraphPad Prism’s Nonliner regression (curve fit) of XY analyses with the formula Y=Bottom+(Top-Bottom)/(1 + 10^((LogEC50-X) ×HillSlope)).

### Affinity Determination

Quantitative analysis of the affinity of humanized antibodies by Bio-Layer Interferometry (BLI) was performed using anti-human IgG Fc capture (AHC) biosensors to immobilize humanized antibody, or using anti-mouse IgG Fc capture (AMC) biosensors to immobilize murine antibody. Antibodies were diluted to 10 μg/mL with PBS containing 0.2% Tween-20, and then abrin were diluted from 200 to 3.13 nmol/L with PBS containing 0.2% Tween-20. Program settings were as follows: samples were washed for 60 s to obtain an initial baseline and made a 180 s association step followed by a 300 s dissociation step. The duration of the loading step was 180 s. Sensors were regenerated with three 5-s pulses of 10 mmol/L glycine-HCl (pH 1.7). The software Data Analysis 9.0 was used to analyze the data. In detail, under the Run Data heading, select Step Correction and Fitting (1:1); click Analyze to generate kinetic data (23). Equilibrium dissociation constant (K*_D_*) indicates affinity between antibody and antigen.

### Cell Viability Test: Inhibition of Abrin-Induced Cell Death

Vero cells (1×10^5^ cell/mL) or Jurkat cells (5×10^5^ cell/mL) were co-incubated with diluted abrin (7 gradients of 3-fold dilution starting at 100 ng/mL or 10 ng/mL, respectively, for Vero cells or Jurkat cells). After 48 h, 10 μL of CCK-8 solution was added to each well and incubated for 4 h at 37°C. Absorbance was measured at 450 nm using a Microtiter plate reader. In follow-up antibody-neutralizing assays, abrin was added at concentrations of 1 or 3 ng/mL to Jurkat cells or Vero cells, respectively. Cells were then incubated with either the presence or absence of diluted antibodies (from 1 μg/mL or 50 μg/mL to 0, respectively). Absorbance was measured at 450 nm using a Microtiter plate reader. The following equation was used to quantify cell viability: Cell survival rate = ((As-An)/(Ac-An)) ×100%; Inhibition rate (%) =((Ac-As)/(Ac-Ab)) ×100%. As: absorbance of samples (cells with medium, CCK-8 solution, abrin and/or antibodies); Ac: absorbance of cell samples (cells with medium, CCK-8 solution but no abrin or antibodies); An: absorbance of blank wells (culture medium and CCK-8 solution only).

### Protein Synthesis in Cell-Free System

The sequence of the Abrin A chain was obtained from UniProt (UniProtKB: P11140.2). A His tag was added to the C-terminus to form the expression plasmid, which was transformed into *E. coli* BL21 (DE3) cells. Abrin A chain was purified from the ultrasonically broken cell lysate by immobilized Ni^2+^ Affinity column chromatography. Different doses of Abrin A chain (from 1 μg/mL to 0.1 ng/mL) were added to the Rabbit Reticulocyte Lysates reaction system (50 μL total reaction system, including total amino acids, reaction buffer, T7 RNA polymerase, RNasin ribonuclease inhibitor and luciferase T7 DNA template) for 90 minutes’ incubation at 30°C. Afterwards, 2.5 μL of the mixed reaction system was mixed with 50 μL of luciferase assay reagent and the fluorescence value was measured immediately at a fluorescence reader. Then Abrin A chain (20 ng/mL) alone or with different concentrations of monoclonal antibodies (from 10 to 1 ng/mL) was added to a rabbit reticulocyte lysate cocktail containing T7 Polymerase, T7 DNA template, etc. in a 0.5 mL microcentrifuge tube and incubated for 90 min at 30°C. The positive control was defined as no addition of Abrin A chain or antibodies to the rabbit reticulocyte lysate cocktail system. The negative control was defined as the addition of Abrin A chain without antibodies to the system. After the incubation, 2.5 μL of the reaction mixture was added to 50 μL of the Luciferase Assay Reagent in luminometer tubes and mixed by pipetting. Luminescence was measured by a luminometer to quantify protein synthesis. The concentration for 50% of maximal inhibitory concentration was defined as IC50. IC50 values were calculated using GraphPad Prism’s Nonliner regression (curve fit) of XY analyses with the formula Y=Bottom+(Top-Bottom)/(1 + 10^((LogIC50-X) ×HillSlope)).

### Fluorescence Activated Cell Scan (FACScan) Analysis

1×10^5^/tube sample Jurkat or Vero cells were harvested and washed twice with FACS solution pre-cooled to 4°C. First, cells were blocked by 10 μL/tube TruStain FcXTM (anti-mouse CD16/32 and anti-Human IgG receptors) for 10 min at RT. Cells were incubated at 4°C for 1 h with 5 μg/mL Alexa-488 labeled abrin alone or with S008 or 10D8 in different concentrations (molar ratios of abrin: S008 or 10D8 were 1:1, 1:10, 1:25, 1:50, 1:100, respectively.). The total volume reached up to100 μL/tube. After incubation, samples were washed twice with pre-cooled PBS and centrifuged at 300 g for 5 min. Cells were fixed in pre-cooled 1% paraformaldehyde fixative for 20 min followed by washing with pre-cooled PBS. Samples were analyzed by FACScan (Becton Dickinson) to compare the fluorescence values of cells treated with labeled abrin with or without S008.

### Laser Scanning Confocal Microscopy Analysis

A 2×10^5^ Vero cells in 1 mL complete DMEM were allowed to adhere to a glass bottom dish (NEST, Cat. No.:801002) at 37°C overnight and then washed twice with pre-cooled PBS at 4°C. A 1×10^6^ Jurkat cells were harvested and washed twice with pre-cooled PBS. All cells were blocked with TruStain FcXTM (anti-mouse CD16/32 and anti-Human IgG receptors) for 10 min at RT and then incubated with Alexa-488 labeled Abrin (0.1 mg/mL) in the presence or absence of Alexa-594 labeled S008 (molar ratio of abrin: S008 as 1:1, 1:10, 1:100) or with Alexa-594 labeled S008 alone for 1 h at 37°C. Cells alone were set as blank controls. Samples were washed twice with pre-cooled PBS, fixed with pre-cooled 4% paraformaldehyde fixative followed by being washed with pre-cooled PBS once. Nuclei were stained with antifade mounting medium containing DAPI for at least 2 min. Cells were visualized and images were acquired using a Zeiss LSM 880 Meta Laser Confocal scanning microscope (Carl Zeiss Foundation, Germany) and analyzed using the Zeiss LSM image browser to visualize the internalized antibody.

### *In Vivo* Protective Experiment in Mice

Animal experiments abided by the guidelines set by the Animal Ethics Committee of Institute of Pharmacology and Toxicology in the strictest form, and the experimental operator obtained the relevant animal experiment qualification certificate. After obtaining approval, experiments were carried out following the 3R principle (Reduction, Re-placement, and Refinement). Intraperitoneal injection of diluted Abrin from 0.25 to 0.125 mg/kg were operated to 6-8-week-old female BALB/C mice (5 mice per group) to observe the mortality to determine the lowest lethal dose (LD). Then a lethal dose of abrin alone or with different concentrations of S008 or 10D8 (from 50 μg/kg to 5 μg/kg) were injected intraperitoneally into 6-8-week-old female BALB/C mice (8 mice per group) simultaneously to investigate the protective effect of the antibodies in a dose-dependent manner. Meanwhile, after injection of a lethal dose of abrin (the injection time of abrin was set as 0 h) into 6-8-week-old female BALB/C mice, a high protective dose of S008 (0.15 mg/kg) was injected intraperitoneally at different time points (at -2, 0, 2, 4, 6, 9, 15, 24 h) to observe the time-dependent protection effectiveness (8 per group). Mice were observed for a minimum of 180 h with preferred observation every 4-8h.

### HE Tissue Staining Analysis

Mice that survived 180 h of observation were euthanized. The heart, liver, spleen, lung, kidney and stomach were removed and the samples were treated with 4% para-formaldehyde, trimmed, dehydrated, embedded, sliced stained with Hematoxylin-Eosin (HE) and sealed according to the tissue staining procedure. All samples were analyzed through microscopic examination. Magnification with 20x×10x and untreated mice were used as controls.

### Data Normalization and Analysis

Each experiment was repeated at least twice. Data analysis was performed using GraphPad Prism version 8.2.1. Data is expressed as average mean ± standard deviation (SD). Statistical analysis was performed by Student’s *t* test or by repeated measures of one-way analysis of variance (ANOVA). Treatments were considered statistically significant when the *P* values < 0.05.

## Results

### Humanization of Anti-Abrin Antibody 10D8

**Figure 1:**
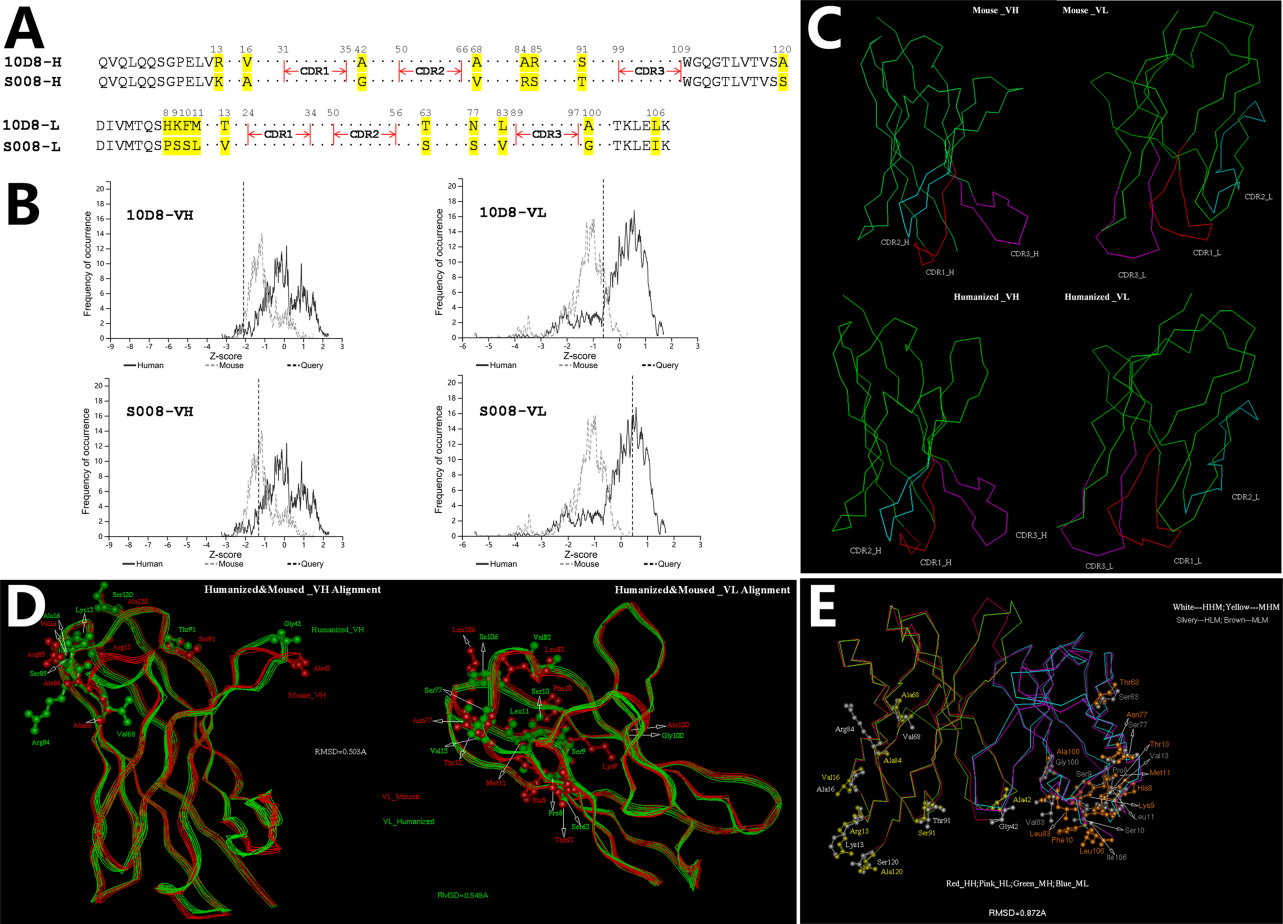
Computer-aided humanization of mouse anti-abrin antibody. **(A)** Amino acid alignment of variable domains between humanized antibody S008 and parent murine anti-body 10D8. **(B)** Humanness degree analysis of variable domain of heavy chain (left) and light chain (right) of 10D8 (upper) and S008 (lower) using http://www.abysis.org. The location of the dotted lines indicates the Z scores. **(C)** Modelled structures of the variable domains of S008 and 10D8 and superimposition of antibody chains; (**C**, Upper left) VH structure of 10D8; (**C**, Upper right) VL structure of 10D8; (**C**, lower left) VH structure of S008; (**C**, lower right) VL structure of S008. **(D)** Superimposition of heavy chain (left) and light chain variable domains (right). **(E)** Superimposition of variable domain of S008 and 10D8.

CDR distribution of the variable domain of heavy and light chain of murine antibody 10D8 were analyzed based on http://www.abysis.org (sequence shown in Chinese Patent 202110764514.4). According to the humanness assessment, the humanized antibody residue sequence aligned with 10D8 (**Figure 1A**). The humanness assessment of the 10D8 variable domain was lower (the Z-score value of heavy chain variable domain was -2.1 and the light chain variable domain was -0.7) than that of S008 (the Z-score values of heavy chain and light chain variable domain were -1.2 and 0.4, respectively) (**Figure 1B**). This indicates that murine antibody 10D8 possesses higher immunogenicity. The designed humanized antibody was named as S008, which showed weaker immunogenicity than 10D8. Based on computer-guided homology modeling, the 3-D variable domain structures of the heavy and light chain of murine and humanized antibody were modeled and optimized (**Figure 1C**). Main chain carbon atom orientation superimposition between 10D8 and S008 was then carried out and root mean square distance (RMSD) of the main chain carbon atoms was calculated. The RMSD value of heavy chain variable domain was 0.503 Å, while that of light chain variable domain was 0.549 Å (**Figure 1D**).

To understand the structural change between the whole variable domain of 10D8 and S008, the whole variable domain (i.e. Fv) conformation was predicted using molecular docking. The 3-D superposition structure between 10D8 and S008 is shown in **Figure 1E**. The whole variable domain main chain carbon atom RMSD value was 0.872 Å; humanized mutations were assigned far from CDRs, which aided in binding to epitopes. These results suggest the humanized antibody S008 should maintain the activity of 10D8.

### Binding Activity of Antibodies to Abrin

**Figure 2:**
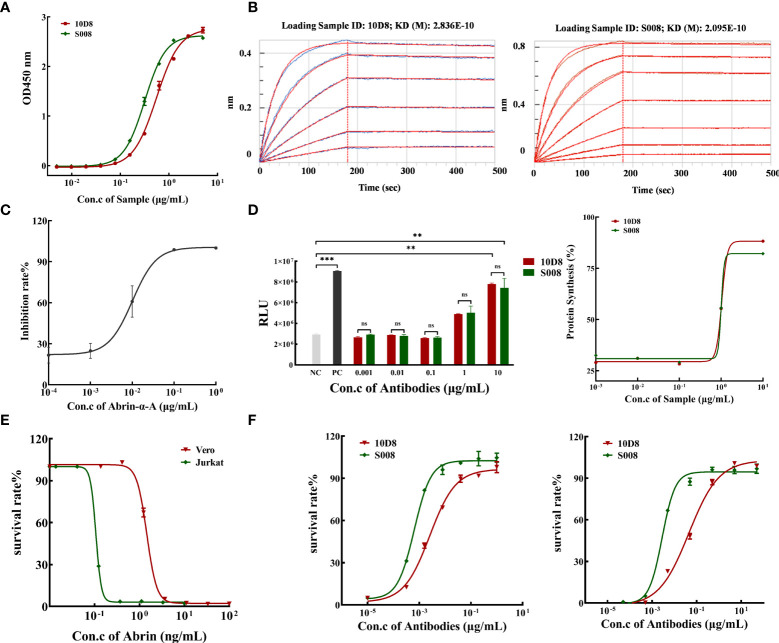
Binding and neutralizing activity of humanized anti-Abrin antibody S008. **(A)** S008 binds to abrin in a dose-dependent manner determined by ELISA. Absorbance values for different antibody concentrations (0.005 to 5 μg/mL) were detected at 450 nm. The EC50 value of S008 was 0.317 μg/mL, while that of 10D8 was 0.553 μg/mL, and EC50 values were calculated using GraphPad Prism’s Nonliner regression (curve fit) of XY analyses; **(B)** Kinetic analysis of antibody binding to abrin measured by the ForteBio method. Left: 10D8 (KD=0.2836 nmol/L); right: S008 (KD=0.2095 nmol/L). **(C)** Inhibition rate of protein synthesis induced by diluted recombinant abrin A chain treatment. The IC50 of protein synthesis *in vitro* was determined to be 0.01 μg/mL; **(D)** S008 and 10D8 neutralized the A chain and recovered protein synthesis in a dose-dependent manner. Left: luminescence values of 10D8 and S008. Positive control without abrin A chain, negative control without antibodies. (**P < 0.01, ***P < 0.001; ns, no significance). Right: Rates of protein synthesis by 10D8 and S008 were calculated using the equation (RLU of the experimental group)/(RLU of positive control group) ×100%. **(E)** Effect of different concentration of abrin on cell death. **(F)** S008 protected both Jurkat (left) and Vero (right) cells against abrin toxicity in a dose-dependent manner.

The purity of isolated antibodies 10D8 and S008 (both IgG1-subtype, kappa, antibodies) were over 95% determined by SEC-HPLC and SDS-PAGE (**Supplementary Figures 1A, B**). ELISA analysis demonstrated that S008 bound to abrin had an EC50 value of 0.317 μg/mL, while that of 10D8 was 0.553 μg/mL (**Figure 2A**). In addition, a ForteBio assay was performed to determine antibody binding kinetics and showed that S008 bound to abrin with the equilibrium KD values of 2.095×10^-10^ mol/L, which was similar to that of 10D8 (2.836×10^-10^ mol/L) (**Figure 2B**), indicating the binding affinities of the two antibodies are comparable. Furthermore, reduced Abrin shown in SDS-PAGE analysis had mainly two bands, Abrin A and B chain (**Supplementary Figure 2A**). We have also proven that S008 was bound to abrin A chain, not B chain, which was similar to 10D8 in western blot assay (**Supplementary Figure 2B**), suggesting that both the epitopes of S008 and 10D8 were on the Abrin A chain.

### Neutralization of *In Vitro* Protein Synthesis and Cell viability Assay

Luciferase assay: rabbit reticulocyte lysate was incubated with different concentrations of abrin A chain in a cocktail containing a mixture of complete amino acids, RNase inhibitor and control luciferase mRNA, for 90 min at 30°C. A luminometer was used to quantify the amount of protein synthesis, and the inhibition rate was equal to (RLU of positive control group minus RLU of the experimental group)/(RLU of positive control group) ×100%. We prepared recombinant abrin A protein to determine the neutralizing effect of the antibodies. The purity of abrin A chain was over 84%, as determined by SDS-PAGE and SEC-HPLC (**Supplementary Figure 3**). Various concentrations of Abrin A chain inhibited luciferase synthesis in a dose-dependent manner *in vitro*, with the IC50 value of 10.09 ng/mL (**Figure 2C**). Neutralizing antibodies 10D8 and S008 was identified to reverse inhibition of protein synthesis (**Figure 2D**, left panel). Synthesis of luciferase in the presence of different doses of S008 is shown in a dose-dependent manner, with the EC50 value of 1.036 and 1.005 μg/mL, respectively (**Figure 2D**, right panel). Abrin is toxic to Jurkat and Vero cells. Abrin will lead to cell death in Jurkat and Vero cells with IC50 values of 0.107 and 1.456 ng/mL, respectively (**Figure 2E**). We established two cell models; neutralizing antibodies 10D8 and S008 were protective for Jurkat (**Figure 2F**, left panel) and Vero (**Figure 2F**, right panel) cells against abrin toxicity in a dose-dependent manner. EC50 values of S008 were 0.642 ng/mL (Jurkat) and 2.826 ng/mL (Vero), respectively. 10D8 was used as the control, with EC50 values of 2.562 and 44.66 ng/mL in Jurkat and Vero cells, respectively, indicating that S008 might possess better neutralizing activity. When the concentration of S008 reaches 100 ng/mL, it can fully protect cells from the toxin. Currently, contrasting to the reported anti-abrin antibodies (e.g. D6F10 or A7C4), S008 seems to possess the best neutralizing potential.

### Toxin Neutralization *In Vivo*

**Figure 3:**
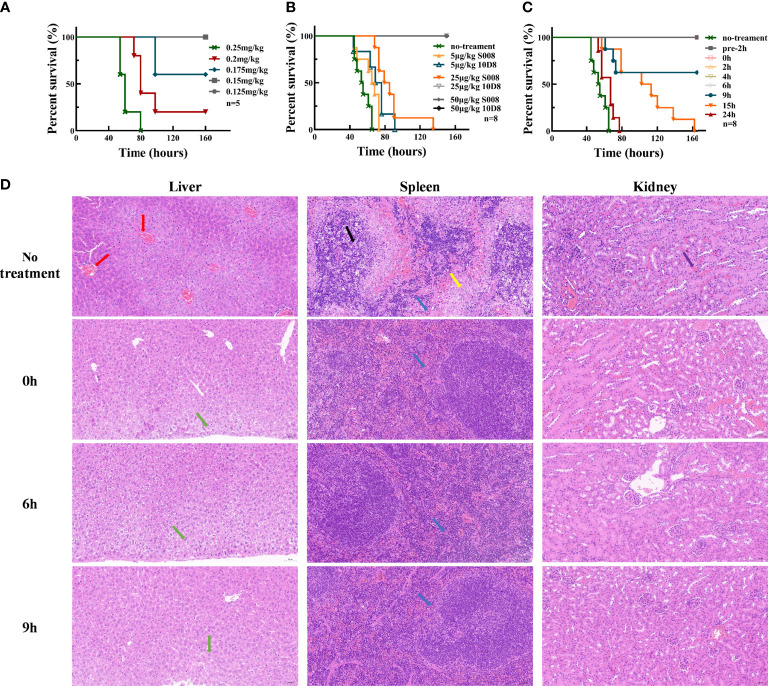
Toxin neutralization by S008 *in vivo*. **(A)** Survival of mice intraperitoneal injected with diluted Abrin (*n*=5). Survival curves drawn using GraphPad Prism version 8.2.1. **(B)** Survival of mice treated with diluted 10D8 or S008 antibodies after challenge with abrin at lethal dose (0.25 mg/kg) (*n*=8). **(C)** Survival of mice administered with 0.15 mg/kg S008 before or after challenge with abrin toxin at lethal dose (0.25 mg/kg) (*n*=8). Post-administration of S008, -2, 0, 2, 4, 6, 9, 15, 24 h time points after abrin injection are noted. When S008 was administered earlier no more than 6 h, mice survived; **(D)** HE-staining of tissues in surviving mice administered 0.15 mg/kg S008 at different time points after abrin injection. Samples were compared with that of mice in the untreated group. From top to bottom: un-treated toxic, 0 h, 6 h and 9 h groups; The left vertical row shows staining of liver, the middle row shows spleen, and the right row shows kidneys. In the no treatment group, venous congestion and dilation (red arrow) were observed in liver, while slight granular degeneration (green arrow) were observed in liver tissue in S008-treated groups, which was normal; meanwhile, there was more desmoplasia (yellow arrow) around the splenic nodules, and more lymphocyte apoptosis and nuclear fragmentation could be seen contrasting to treatment groups (black arrow). There was a small amount of neutrophil infiltration (blue arrow) in all groups; Ecchymosis (purple arrow) in the renal cortex and medulla could be seen in toxic but not in antibody treated mice. Magnification is 20x×10x.

Experimental data showed that 0.25 mg/kg abrin caused toxic shock and killed 100% of mice as lethal dose (LD) (**Figure 3A**). As shown in **Figure 3B**, all mice injected with lethal dose of abrin (0.25 mg/kg) and 50 μg/kg S008 survived, while those injected with 25 μg/kg 10D8 group also survived abrin exposure. Therefore, S008 appeared to have a weaker neutralizing capacity than 10D8 (*n*=8), which might be due to the *in vivo* antigenicity of human Fc region of S008 in mice. **Figure 3C** shows the time-dependence of the protective efficacy of S008 against poisoning death induced by abrin. Preventive injection of higher doses of S008 (150 μg/kg) protected mice against abrin, not only in “pre-2h” group (injection of S008 was 2 h prior to intraperitoneal injection of abrin; “pre-2h” group in **Figure 3C**), but also in post-injection groups. Post-administration data of S008 at 0, 2, 4, 6, 9, 15, 24-h time points after intraperitoneal injection of abrin suggest that S008 injection protects against the lethal effects of abrin. When the time to rescue was 6 h or less, the survival rate of mice was 100%. In the 9-hour group, the survival rate of mice was 60%, indicating that the “effective time” of S008 to treat Abrin poisoning might be greater than 6 hours, indicating that after exposure, there would be at least 6 hours to seek treatment. Meanwhile, although the efficacy of treatment with S008 given 15 h after Abrin injection seemed not satisfactory, the survival time of the mice in the 15 h group was longer than that of the 24 h group, suggesting the potential protection efficacy of S008 even in later stages after poisoning. According to the HE staining shown in **Figure 3D**, S008 effectively protected organs from obvious abnormality. The liver, spleen and kidney, especially, compared with the toxic group, S008 groups (regardless of 0 h, 6 h, or 9 h post-exposure) showed less inflammation. Venous congestion and dilation could be seen in the livers of the toxic group, as well as extensive watery degeneration of hepatocytes around the central vein. There was also decreased splenic nodules, greater desmoplasia around the splenic nodules and more lymphocyte apoptosis. In the kidney, cortical and medullary ecchymosis could be seen in the toxic group. However, except for mild neutrophil infiltration, most acute symptoms were either not observed or weakened in the antibody-treated groups. It is worth mentioning that there was no significant difference between the 6 h and 9 h groups in comparison with 0 h, suggesting that the antibody has a relatively long duration of effective protection.

### S008 Does Not Prevent Abrin From Binding Cells or Endocytosis

**Figure 4:**
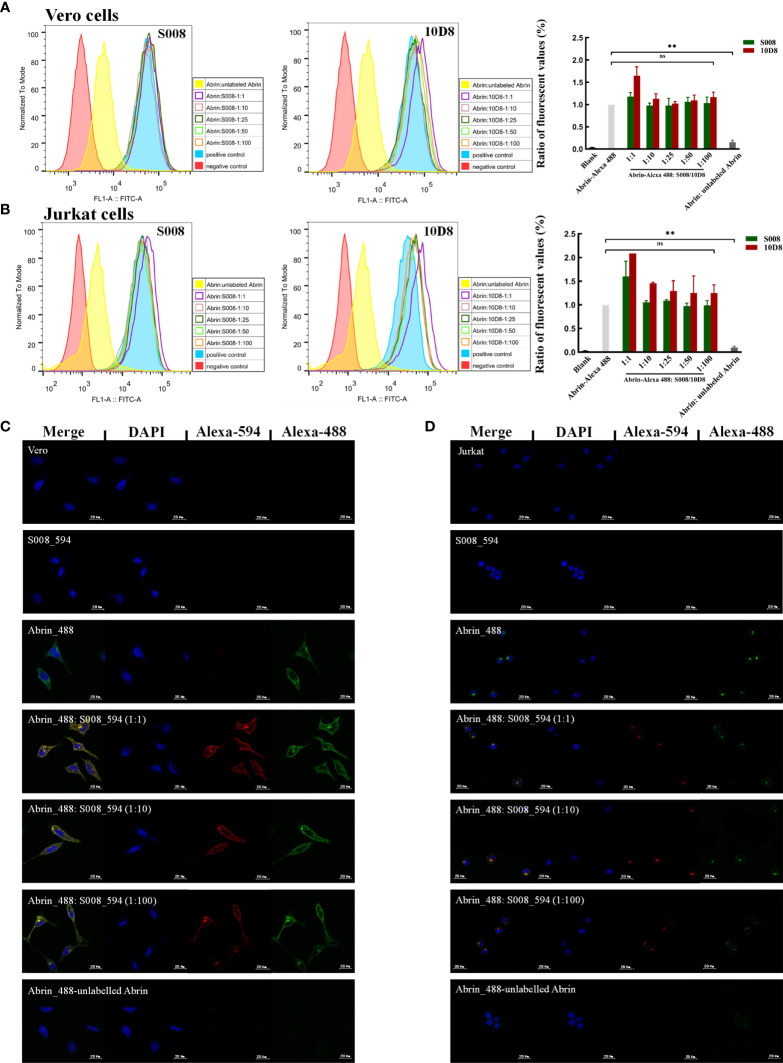
S008 does not inhibit Abrin binding or entering Jurkat or Vero cells. **(A, B)** FACS analysis of the ability of S008 and 10D8 to block Abrin binding to Jurkat or Vero cells. Intracellular fluorescent signals of fixed cells were analyzed using a FACScan system. The ratio of the fluorescent values of experiment group and positive control group are show. (**P < 0.01; ns, no significance). **(C)** Confocal microscopy analysis of S008. **(D)** Confocal Microscopy analysis of S008. Abrin was labeled with Alexa-488 and S008 was labeled with Alexa-594. Nuclei were stained with DAPI. Images were acquired using Zeiss LSM 880 Meta Laser Con-focal scanning microscope and analyzed using the ZEISS ZEN 3.4.

S008 was raised as an inhibitory antibody against the cytotoxic effects of abrin. Here, we analyzed the binding of Alexa-488–labeled abrin to Jurkat or Vero cells in the presence or absence of monoclonal antibody S008. FACS analysis and fluorescence microscopy results indicated that S008 does not prevent binding of abrin to the cell surface, even at high concentrations. Only superimposed peaks could be observed when cells were incubated with labeled abrin in the presence or absence of S008 (**Figures 4A, B**), suggesting that S008 was unable to inhibit abrin binding to cells, while the control antibody 10D8 did. As shown in **Figures 4A, B** (right panel), the ratio of the fluorescent values of the antibodies group compared to the positive control group (cells incubated with only labeled abrin) was about 1, indicating that S008 did not inhibit binding, entry or adsorption of abrin the Jurkat or Vero cells. Confocal microscopy showed internalization of Alexa-594 labeled S008 together with abrin into Jurkat or Vero cells, indicating the S008-abrin complex could be internalized with the help of the abrin B chain, suggesting the neutralizing effect of S008 might happen intracellularly (**Figures 4C, D**).

## Discussion

Abrin is a highly toxic protein toxoid that causes lethal poisoning reactions and toxic shock. As a potential biological weapon, Abrin can be easily prepared and used with hostile intent in terrorism. It was reported that Abrin is 70 times more toxic than Ricin and 2885 times more toxic than VX nerve agent (24). The lethal dose of Ricin was 1-15 μg/kg for inhalation or injection and 1-20 mg/kg for the ingestion method under different exposure conditions (11, 25). However, in our study we found that the LD value *in vivo* was higher compared to the reported data. This might be firstly due to the diversity between different plant strains. The Abrin protein we obtained was purified from the natural plant seeds of *A. precatorius*. Secondly, this kind of abrin might be a mixture, which also contained several kinds of weaker lethal subclass toxins such as Abrin-b and/or Abrin-c.

In 2020, a suicide case was reported in Arizona, USA, by injecting *A. precatorius* seeds powder intramuscularly or subcutaneously. The patient was admitted to hospital 17.5 h after the toxin injection and died of multiple organ failure after 4 days of supportive treatment (9). Currently, there is no vaccine or therapeutic drug candidates for abrin poisoning. Considering the virulence of Abrin and the need for urgent treatment, monoclonal antibodies have become a preferred choice for the treatment of abrin poisoning due to their high specificity and minimal side effects. A series of research works (12, 20, 21) introduced that D6F10, A7C4 and a set of chimeric antibodies (RB9/RB10/RB28/RB30) acted effectively as neutralizing antibodies. However, there is no declaration of any therapeutic antibody against Abrin that can be used even in pre-clinical research stage. Therefore, we planned to obtain a humanized antibody capable of treating Abrin poisoning in humans as a matter of urgency.

In a previous study, we obtained a mouse anti-abrin monoclonal antibody, named as 10D8, using hybridoma technology. The antibody 10D8 possesses high affinity and protective function tested in *in vitro* cell model and in *in vivo* mouse poisoning model. In the present research study, a novel humanized antibody named as S008 was obtained by using CDR grafting and computer-guided structure modeling method in an effort to decrease immunogenicity. The Z-value could be seen as a quantitative indicator of the immunogenicity, the higher the Z-value, the weaker the immunogenicity to human beings (**Figure 1B**). Based on the 3-D crystal structure of abrin-A (PDB code: 1ABR), the potential epitopes of S008 were distributed widely around the whole molecule theoretically. The potential binding domain between the antigen (i.e. abrin-A) and antibody (10D8 or S008) were complex and difficult to determine using the molecular docking method. Therefore, to analyze the potential function of S008, the structural comparison between S008 and its parent 10D8 was also analyzed (**Figures 1C–E**), and consequently the theoretical results showed that the humanization of 10D8 to S008 should remain the potential function similar as 10D8.

In this study, we verified that antigen-binding activities of S008 were similar to the parent 10D8 antibody by ELISA and ForteBio experiments. Meanwhile, the detoxification function of S008 was similar or better than 10D8 in *in vitro* neutralizing assays. Notably, in *in vivo* protection assay, both S008 and 10D8 showed good protective effects for poisoned mice; however, the protection effect of S008 seemed slightly weaker. Based on these results, we speculated that the weaker neutralization activity of S008 might be due to the existence of the human Fc region, which could cause an accelerated immune clearance rate *in vivo*. Despite this, S008 showed better therapeutic protection than 10D8. Compared with another neutralizing anti-SEB antibody reported previously (26), S008 had a longer therapeutic window for efficient treatment, suggesting ample time to acquire S008 after exposure. It was also suggested a longer therapeutic window period than the reported data of the chimeric antibodies, e.g. RB10, RB28, against Abrin intoxication (21).

It is well established that binding of abrin to the galactose receptor on the cell surface is the first event in abrin-mediated toxicity. When abrin enters the cells, the A chain is separated from the B chain and mediates ribosomal inhibition. While anti-A chain antibodies have been developed, their mechanisms remain unclear. Here, FACS analysis and fluorescence microscopy showed that anti-abrin A chain antibody S008 does not prevent the abrin toxin from entering the cells, which was similar to the mAb A7C4 reported previously (20). This suggests that the epitope of S008 binding to Abrin subunit A chain was far from the B chain, and S008 should not prevent abrin from binding to the membrane receptors. The inhibitory role of S008 might instead be mediated by binding to the toxic sites of abrin, which requires further tests, or preventing intracellular separation of the A and B chains, or directly inducing lysosomal degradation of abrin-S008 complexes.

## Conclusion

In summary, abrin is an easily purified potent high-risk toxin. The need for specific humanized antibodies with neutralizing activity is urgent. This work identified a high-affinity abrin-specific antibody S008, which was capable of neutralizing abrin both *in vitro* and *in vivo* with low EC50 values. S008 should be further explored as an ideal candidate for the effective treatment of abrin poisoning.

## Data Availabillity Statement

The original contributions proposed in the research are included in the article/**Supplementary Material**. Further inquiries can be directed to the corresponding author.

## Ethics Statement

This animal study was reviewed and approved by the Institutional Animal Care and Use Committee of Academy of Military Medical Sciences.

## Author Contributions

JP, JWu, and NS contributed equally to this research project and to data acquisition. JP contributed to the conception and wrote the first version of the manuscript. HX, LL, JWa, XL, HX, JF and XL provided methodological support. LC and CQ conceived and guided the study. All authors contributed to the article and approved the submitted version.

## Funding

This study was supported by a National Natural Sciences Foundation of China (grant 31771010).

## Conflict of Interest

The authors declare that the research was conducted in the absence of any commercial or financial relationships that could be construed as a potential conflict of interest.

## Publisher’s Note

All claims expressed in this article are solely those of the authors and do not necessarily represent those of their affiliated organizations, or those of the publisher, the editors and the reviewers. Any product that may be evaluated in this article, or claim that may be made by its manufacturer, is not guaranteed or endorsed by the publisher.
