# Supplementary material for: A Novel Humanized Anti-Abrin A Chain Antibody Inhibits Abrin Toxicity In Vitro and In Vivo
Source: Front Immunol. 2022 Feb 4;13:831536. doi: 10.3389/fimmu.2022.831536 (PMC8855095; doi:10.3389/fimmu.2022.831536)
Supplement: Supplementary file 1 [file DataSheet_1.docx]

# SUPPLEMENTARY MATERIAL


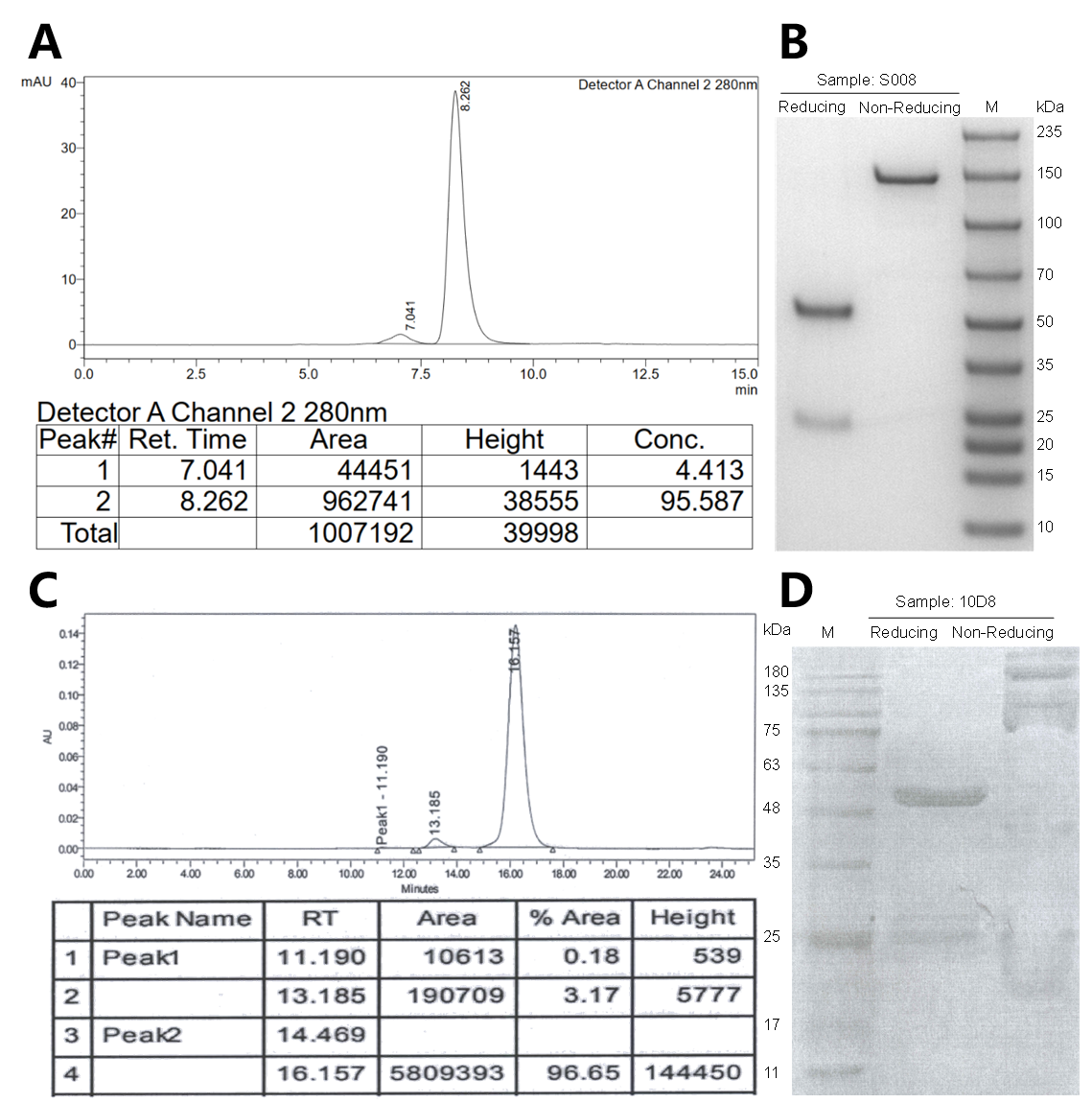


Supplementary figure 1. Antibodies purification assay of SEC-HPLC and SDS-PAGE. (A) SEC-HPLC result of antibody S008; (B) SDS-PAGE result of antibody S008. (C) SEC-HPLC result of antibody 10D8; (D) SDS-PAGE result of antibody 10D8. M: Maker.


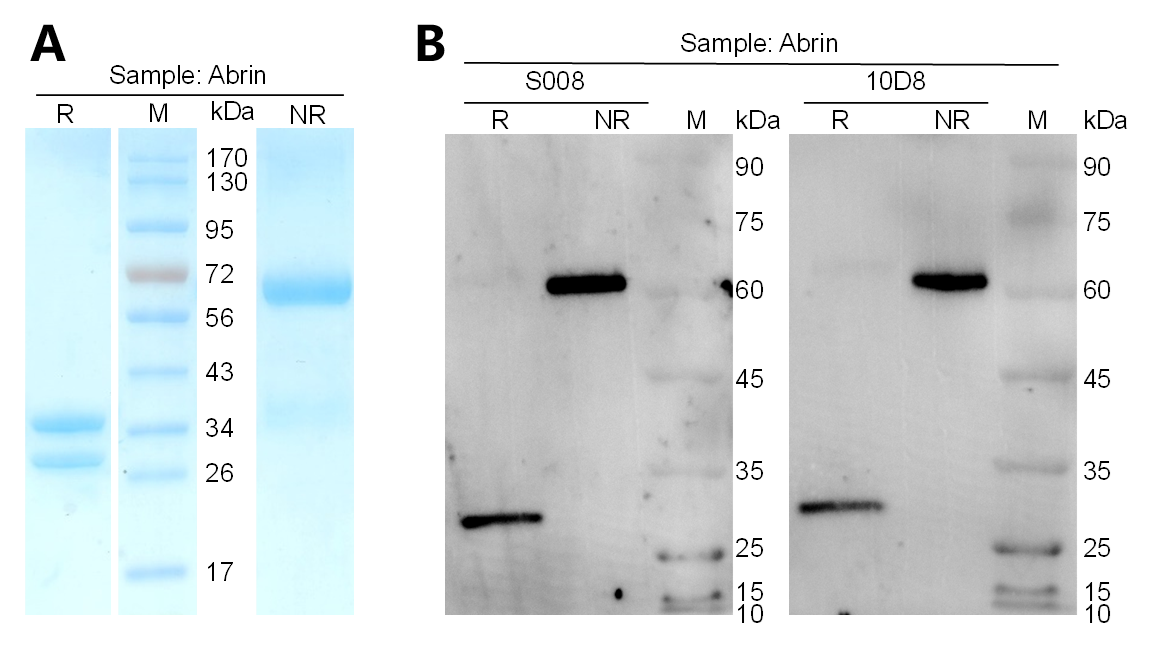


Supplementary figure 2. Antibodies bound to Abrin A chain. (A) SDS-PAGE result of Abrin with reducing agent (R) and non-reducing agent (NR), respectively. Abrin is about 63 kDa, and Abrin A chain is about 30 kDa. (B) Western blot result of antibodies binding to reducing (R) and non-reducing (NR) Abrin. M: Maker.


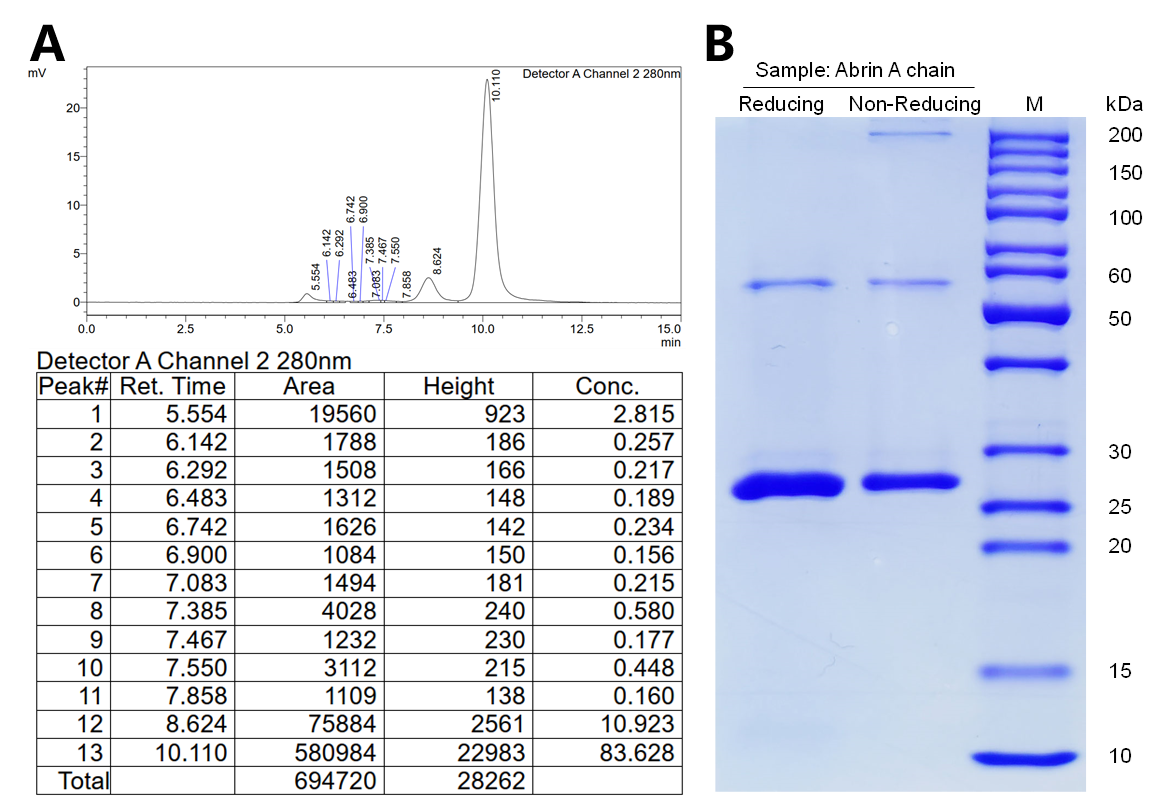


Supplementary figure 3. Abrin A chain purification assay of SEC-HPLC and SDS-PAGE. (A) SEC-HPLC result of Abrin A chain; (B) SDS-PAGE result of Abrin A chain. M: Maker.
